# Environmental complexity: A buffer against stress in the domestic chick

**DOI:** 10.1371/journal.pone.0210270

**Published:** 2019-01-14

**Authors:** Irene Campderrich, Franco Nicolas Nazar, Anette Wichman, Raul Hector Marin, Inma Estevez, Linda J. Keeling

**Affiliations:** 1 Department of Animal Health, Neiker-Tecnalia Basque Institute for Agricultural Research and Development, Vitoria-Gasteiz, Spain; 2 Department of Animal Environment and Health, Swedish University of Agricultural Sciences, Uppsala, Sweden; 3 Instituto de Investigaciones Biológicas y Tecnológicas, Consejo Nacional de Investigaciones Científicas y Técnicas, Universidad Nacional de Córdoba and Instituto de Ciencia y Tecnología de los Alimentos, Facultad de Ciencias Exactas, Físicas y Naturales, Universidad Nacional de Córdoba, Córdoba, Argentina; 4 IKERBASQUE, Basque Foundation for Science, Bilbao, Spain; Tokat Gaziosmanpasa University, TURKEY

## Abstract

Birds kept in commercial production systems can be exposed to multiple stressors from early life and this alters the development of different morphological, immunological and behavioural indicators. We explore the hypothesis that provision of a complex environment during early life, better prepares birds to cope with stressful events as well as buffers them against future unpredictable stressful episodes. In this study, 96 one day old pullets were randomly distributed in eight pens (12 birds/pen). Half of the chicks (N = 48) were assigned to a Complex Environment (CENV: with perches, a dark brooder etc.) the others to a Simple Environment (SENV: without enrichment features). Half of the birds from each of these treatments were assigned to a No Stress (NSTR, 33°C) or to an acute Cold Stress (CSTR, 18–20°C) treatment during six hours on their second day of life. At four weeks of age, chicks with these four different backgrounds were exposed to an Intermittent Stressful Challenges Protocol (ISCP). In an immunological test indicative of pro-inflammatory status Phytohemagglutinin-P (PHA-P), the response of CSTR birds was ameliorated by rearing chicks in a CENV as they had a similar response to NSTR chicks and a significantly better pro-inflammatory response than those CSTR birds reared in a SENV (five days after the CSTR treatment was applied). A similar better response when coping with new challenges (the ISCP) was observed in birds reared in a CENV compared to those from a SENV. Birds reared in the CENV had a lower heterophil/lymphocyte ratio after the ISCP than birds reared in SENV, independently of whether or not they had been exposed to CSTR early in life. No effects of stress on general behaviour were detected, however, the provision of a CENV increased resting behaviour, which may have favoured stress recover. Additionally, we found that exposure to cold stress at an early age might have rendered birds more vulnerable to future stressful events. CSTR birds had lower humoral immune responses (sheep red blood cells induced antibodies) after the ISCP and started using elevated structures in the CENV later compared to their NSTR conspecifics. Our study reflects the importance of the early provision of a CENV in commercial conditions to reduce negative stress-related effects. Within the context of the theory of adaptive plasticity, our results suggest that the early experience of the birds had long lasting effects on the modulation of their phenotypes.

## Introduction

Birds used in poultry production systems can be exposed to multiple stressors early in life such as hatching without maternal contact, transport, heat or cold environmental temperature [1–3]. Understanding the impact that these early stressors have on the birds’ behaviour and physiology is important from an animal welfare perspective and could, at the same time, elucidate the evolution of phenotypic diversity in populations [4]. In this context, the adaptive plasticity theory proposes that some of the inputs animals receive during their development, perhaps the stressors to which they are submitted or the conditions of their rearing environment, are factors capable of producing lasting alterations. These alterations may, in turn, lead to the development of a certain phenotype that could be adaptive for the individual´s future life. It can, for instance, provide individuals with advantages to face future challenges. On the other hand, it can result in the development of a phenotype with deleterious effects on fitness, perhaps making individuals more sensitive to future stressful conditions [5].

Koolhaas et al. [6] state that the term ‘stress’ should be restricted to conditions where the environmental demand exceeds the natural regulatory capacity of an organism. Accordingly, the stressor is defined as a stimulus or environmental condition that induces such a state. This definition of stress was chosen for its simplicity and because it helps in the delimitation of the stress concept. This definition also integrates two aspects of interest for the expression of stress: unpredictability and uncontrollability which will be considered in the development of the present study.

Not all stressors induce the same response and not all individuals cope with stress in the same manner. In fact, among the most important factors determining the stress response are those referring to the individual per se [7, 8] like the birds’ genotype, previous experience or health status. Individual differences may explain why a stressor has a profound effect in some birds while having little or no effects in others. Factors such as the nature, intensity, frequency and duration of the stressor also influence its consequences [7, 9, 10] and effects may be different when stressors are applied alone or combined with others [10]. When stressors are repeated or are sustained in time, physiological changes may affect diverse body systems. For example, the immune system is influenced by stress response mediators such as corticosterone [11,12], whose immunosuppressive effects could reach both to the humoral and cellular components of immunity affecting the efficacy of the responses that characterise this system. Examples of stress induced suppression include; lower relative weights of the thymus and spleen in broilers [13], a reduction of the Phytohemagglutinin-P (PHA-P) response and of the capacity to produce antibodies against sheep red blood cells (SRBC), together with an elevation of the heterophil/lymphocyte (H/L) ratio in Japanese quail [14]. A rapid apoptosis in immature T- and B-cells induced by glucocorticoids has also been observed in White Leghorn chickens [15]. Stress may have other behavioural [16,17] and performance related [18] deleterious effects, which can lead to serious problems or even economic losses for producers [19, 20]. At the behavioural level, broilers showed higher fear responses following mechanical restraint than their non-stressed conspecifics [16] and white Leghorn chicks were more inactive following an E-coli injection compared to chicks injected with an innocuous saline solution [17]. Regarding performance parameters, broilers exposed to heat or cold stress showed reduced feed intake and body weight gain, and an increased feed conversion rate compared to birds reared in thermoneutral conditions [18]. These birds also had lower antibody titres against SRBC, indicating a lowered humoral immunological response. Understanding the impact of short and long-term stressors to which young chicks can be exposed in their productive life and exploring the potential to reduce their impact are highly relevant to promote birds´ resilience and thus welfare and performance.

The welfare benefits derived from increasing environmental complexity, for example, by the use of artificial structures such as perches or cover, have been widely documented in poultry [21–29]. Providing production animals with opportunities to interact in a complex environment may help them to express more of their natural behaviour [21] and counteract some stress related deleterious effects [14, 30]. The red jungle fowl, ancestor of the domesticated chicken [31], inhabits forests or areas with vegetative cover [32] which provides them with opportunities to explore the three-dimensional environment. Moreover, for prey species, the opportunity to take cover or jump onto perches or platforms may be considered a form of controlling their surrounding environment [33]. Such control is probably associated with a positive emotional state of security/safety and it is well documented that controllability helps animals cope better with stress challenges (see Koolhaas et al. [6], for an extended review).

According to the predictive adaptive response hypothesis, a part of adaptive plasticity theory, the environment that individuals encounter in the early stages of their development will determine their phenotypic plasticity and consequently affect their fitness [34]. The humoral and cellular immune responses of quail reared in complex environments were stronger than those from birds reared in barren environments [14]. Laying hens reared in complex environments also showed better spatial skills than those reared in barren environments [35] and thus could be expected to adapt better to new environments. Taking these into consideration, we propose that providing birds with a complex environment early in their development will increase their plasticity at the immunological and behavioural level, thus better preparing them to confront the challenges they may encounter later in life. We explore the hypothesis that provision of a complex environment during early life prepares birds to cope better with stressful events or buffers them from future unpredictable stressful episodes. Additionally, we investigate whether controlled exposure to early stress increases birds´ resilience or whether it actually amplifies their sensitivity to future challenges.

In the present study, we worked with an integrated model that allowed us to identify the consequences of acute and unpredictable stressful challenges in domestic chicks reared in simple or complex environments (SENV or CENV respectively). We predicted that experiencing a CENV would attenuate the impact of acute stress and improve birds’ ability to cope with future unpredictable challenges. If these predictions are confirmed, it would be possible to develop protocols for the improvement of birds’ resilience to stress, thus having a positive impact on their welfare and performance.

## Material and methods

### Animals, transport and housing conditions

One hundred and four domestic fowl chicks (*Bovan robust*, a white layer strain) were purchased from a Swedish hatchery (Swedfarm AB, Linköping) in June 2014. The chicks were collected the day of hatching and immediately transported to our experimental facilities at Lövsta (Swedish University of Agricultural Sciences). The experimental room contained eight (1.2 x 1.2 m) pens built with a wooden structure and wire mesh. Visual contact across neighbouring pens was avoided by placing 97 cm high wooden barriers between the pens. The birds were fed *ad libitum* with a standard commercial diet, designed according to their rearing phase. Feed and water were provided in round shaped feeders (12 cm/ pullet) and bell drinkers (four cm/pullet) and wood shavings were used as litter. The room had automatically controlled photoperiods, temperature and ventilation that were adjusted during the rearing according to the recommended commercial practices for pullets [36].

Upon arrival, all chicks were individually weighed (Sartorius BL 1500 measuring to the nearest 0.1 g) and white numbered leg rings were placed on both legs for individual identification. Birds were ranked on day two according to body weights and were distributed across eight groups of 13 chicks in a balanced manner to provide similar mean weights for each group. Even though the whole study was performed with 96 pullets and experimental final groups consisted of 12 birds, we placed an extra chick in each group for the first week to compensate for possible early mortalities. After that time, in groups where no deaths occurred, the extra chick was removed and placed in an extra pen in the same room. These birds were used in additional pilot studies and then at the end of the third week they were given away to private homes.

At eight days of age birds were tagged with two, 3 cm diameter, white laminated round-shaped paper tags attached by a thin plastic filament through the skin of each wing [23]. The tags had numbers printed on both sides to facilitate visual individual identification. Leg rings were removed at three weeks of age.

### Ethical note

This project was performed according to the ethical requirements regarding animals used for scientific purposes established in Sweden. The experimental protocol was approved by the Uppsala Ethical Committee (ethical permit number C70/14). The humane endpoints used in this study were any bird losing more than 10% of its bodyweight, having any large injury or being sick and unable to stand normally. Birds were checked twice per day and weighed each week. Birds reaching any of these endpoints were euthanized. Euthanasia was by dislocation of the neck, which was preceded by stunning. In addition to the pre-determined endpoints, during the study birds showing any type of sickness behaviour were monitored regularly throughout the day and their situation discussed with the responsible veterinarian. If birds did not start to show signs of recovery they were euthanized. Birds that started eating, drinking and moving around the pen were considered to be recovering.

### Experimental design

Cold stress (CSTR) was used in this experiment as an acute environmental stressor to simulate a stressful situation that chicks may experience during transport from the hatchery to the rearing facilities. It is known that thermoregulation in chicks is underdeveloped the first ten days after hatching [37], which makes them especially vulnerable to cold stress [38]. Birds from stressed (CSTR) and non-stressed (NSTR) groups were reared in complex and simple environments (CENV and SENV, respectively) to examine the potential short and long-term buffering effects of the complex environment on birds´ morphology, immunology and behaviour. In addition, birds were exposed to an Intermittent Stressful Challenges Protocol (ISCP) with the goal of assessing birds’ coping ability according to their early life experiences. The ISCP consisted of exposure to stressors applied at different hours and different days during a week. Uncontrollable and unpredictable events have been described as major sources of stress in rodents (reviewed in Koolhaas et al. [6]) and unpredictability may also significantly affect learning abilities and long-term behaviour in domestic fowl [39,40].

**Fig 1:**
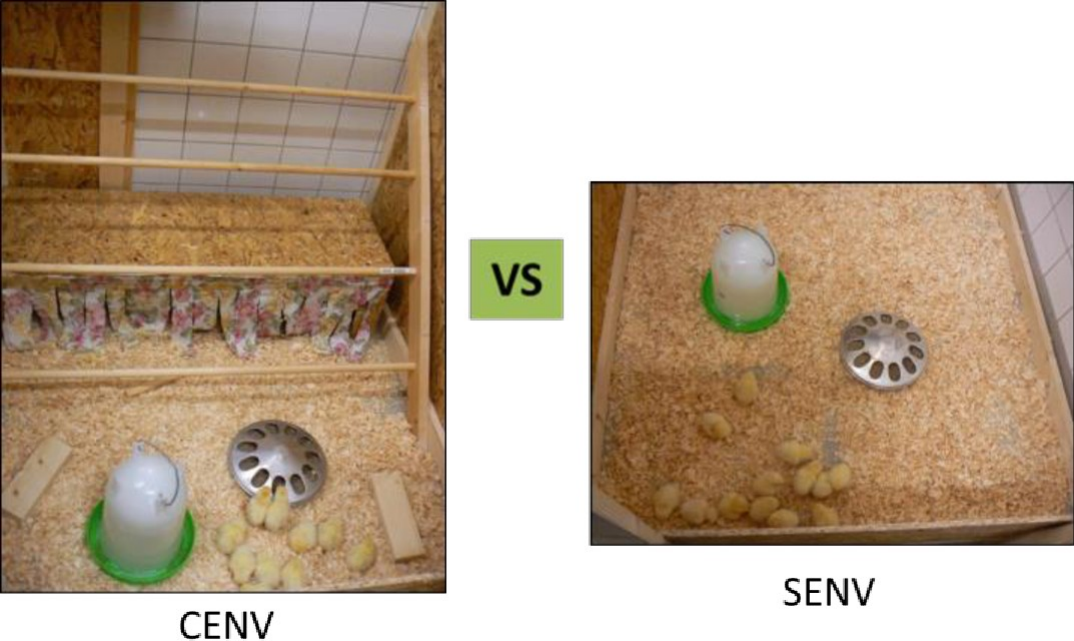
Complex versus simple environments. Photo of two pens from the current experiment; the one on the left side represents the Complex Environment (CENV) treatment provided with a drinker, a feeder, wood shavings, three wooden blocks, four perches at different levels and a hide area; while the one on the right side represents the Simple Environment (SENV) treatment, provided only with a drinker, feeder and wood shavings.

A two-factorial design combining the effects of stress and environmental complexity was used. Half of the birds (48) were assigned to either a SENV or a CENV treatment. The SENV housing contained wood shavings, a feeder and a drinker. The CENV had the same features but, in addition, the birds had access to small solid blocks (10 x 25 x 5 cm; W x L x H), round perches (110 cm x 2.5 cm; L x diameter) at four levels (15, 32, 52 and 66 cm; H) and a hide area (40 x 110 x 20 cm; W x L x H). All the structures were constructed of wood. The hide could be used as a dark area to rest in and the roof could be a platform to roost on. See Fig 1 for a detailed description of the SENV and CENV treatments.

**Fig 2:**
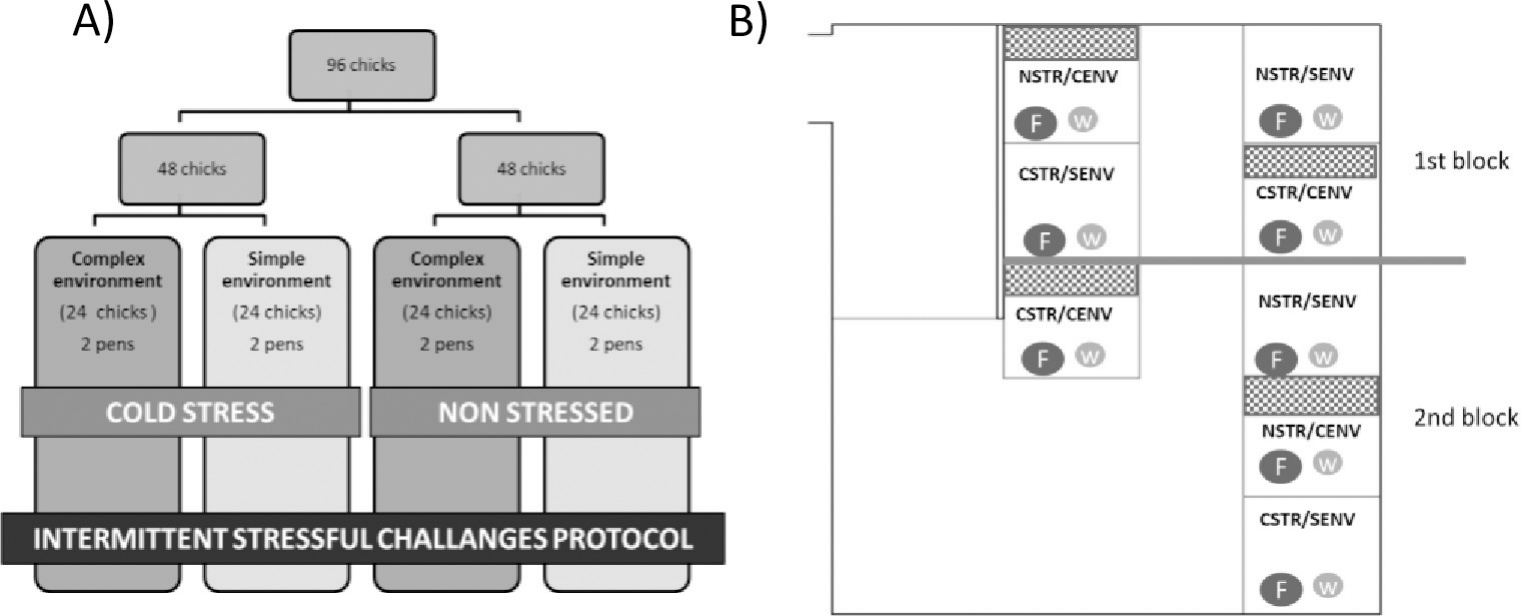
Experiment description. (A) *Experimental design*: for this experiment 96 domestic fowl chicks (*Bovan robust*, a white laying strain) were randomly assigned to either a Complex or a Simple home environment (CENV, SENV respectively; N = 48 per treatment). Additionally, birds from each environment were allocated to either a Cold Stress (CSTR) or No Stress (NSTR) treatment. At four weeks of age birds with different early life experiences (CSTR-CENV, CSTR-SENV, NSTR-CENV, NSTR-SENV, two pens/treatment, eight pens in total) were exposed to an Intermittent Stressful Challenges Protocol (ISCP); (B) *Distribution of the treatments across the experimental room*: each square division represents a pen, F indicates the feeder, W the drinker, CENV refers to Complex Environment, SENV to Simple Environment; NSTR refers to a No Stress group while CSTR refers to a Cold Stress group.

On day two, half of the chicks from each environment were assigned to a CSTR treatment while the other half were allocated to the NSTR treatment. For this, all chicks from each pen were collected and were placed in pairs in a compartmented cardboard box (box size 40 x 60 x 15 cm; compartment size 20 x 15 x 15cm; W x L x H). Boxes with CSTR birds were kept in a room under suboptimal temperatures of 18–20°C during six hours. NSTR boxes were kept at a standard temperature of 33°C. To ensure chick welfare during the treatment exposure and to verify that chicks were indeed CSTR, we monitored rectal temperatures with a digital thermometer (DocMorris CE 0123) introduced one cm into chicks´ cloaca every two hours. After the stipulated six hours, birds were returned to their home pens. The locations of the pens with birds allocated to the environmental (CENV and SENV) and stress (CSTR or NSTR) treatments were balanced in the room and split into two blocks (See Fig 2A and 2B for a detailed description of the experimental set up and distribution of the experimental groups).

To test how pullets with different early life experiences cope with new challenges, they were exposed to an Intermittent Stressful Challenges Protocol (ISCP) at four weeks of age. The ISCP used in the present study was applied during five consecutive days, from 23 to 27 days of age, and multiple uncontrollable and unpredictable stressors applied to the entire room were used. The stressors consisted of (1) random normal commercial practices, such as modification to the heating system (day 23 of age), changing the feeders and drinkers for bigger ones and cleaning the pens, which included removing and replacing the bedding material (day 27 of age); (2) one hour exposure to heavy metal music played at 90dB, all five days in a row at different times of the day; (3) daily exposure to unpredictable loud noises (reproduced also at 90dB), which included recorded sounds of predators or other animals and mechanical sounds of trains, airplanes or ambulances, and (4) changes in the timing of the light/dark phases by programming the lights to switch on and off at random intervals during the last three days (day 25 to 27 of age).

In order to explore the reaction of the pullets to the ISCP, every second day during the exposure to heavy metal music, we registered every 15 min the number of chicks that were feeding. We started our observations 30 min before the music was switched on and stopped 30 min after it was switched off.

#### Dead birds

Only three birds from three different treatments had died by the end of the first week. The extra chicks in those groups were used to complete the experimental group, while extra chicks in other groups were removed and placed in an extra pen as described previously. No additional mortalities were observed until the sheep red blood cells (SRBC) injection at four weeks of age. The first deaths were detected during the morning check the day after the injection (12 hours after injection). Bacterial contamination of the injected blood is a possible explanation for these mortalities, but we cannot confirm this. A total of 24 chicks either died (n = 11) or were euthanized (n = 13). The mortalities were an unfortunate and unexpected event following this routine challenge procedure. Some chicks were euthanized immediately, some sick birds recovered and the time interval between the injection until euthanizing the last of the non-recovering birds was 2 days. Criteria for the decision of when to euthanize is described in the ethical note section. The mortalities are shown in detail in Table 1. Since there was no longer any possibility to replace birds, all subsequent statistical analyses were corrected for the different numbers of birds in the pens. The mortalities resulted in differences in stocking density and resource allocation between pens for the remainder of the study.

**Table 1:** Dead and euthanized birds after the injection of SRBC. The first column indicates the pen number; the second column indicates the corresponding treatment (CSTR/NSTR referring to the Cold Stress or No Stress treatments and SENV/CENV referring to the Simple or Complex Environments respectively); the last column lists the identity numbers of the birds in the pen; the birds that died are marked in dark grey and those that had to be euthanized are marked in light grey.

Pen N°	Treatment	Birds Id											
1	CSTR/CENV	84	80	71	81	77	60	22	5	75	46	58	8
2	CSTR/SENV	89	28	38	24	37	101	54	65	25	12	4	6
3	NSTR/CENV	41	95	88	49	44	76	32	18	13	50	78	48
4	NSTR/SENV	97	26	31	43	2	90	42	27	14	85	91	96
5	CSTR/CENV	45	59	21	70	29	10	61	57	82	53	63	79
6	NSTR/SENV	20	86	68	83	30	35	3	73	64	7	51	15
7	NSTR/CENV	72	69	92	23	74	39	102	87	52	47	17	9
8	CSTR/SENV	66	16	55	33	94	19	34	36	56	1	11	93

#### Morphological measures

All birds were individually weighed (Sartorius BL 1500 measuring to the nearest 0.1 g) once per week during the experiment (weeks one to six). The increase in grams per week was used to calculate growth rate. At the end of the experiment (week six), tarsus length and width were measured following the procedure described by Campo and Prieto [41] with a digital calliper (Biltema, Sweden) to the nearest 0.01 mm. Body weight and tarsus measures were analysed separately but also combined to calculate body condition (calculated as the ratio: body weight/tarsus length). Relative fluctuating asymmetry (RFA) was calculated as the absolute difference between the right and left legs divided by the mean between the left and right measures [42,43]. These morphological indicators were selected because of their previously documented association to measures of individual quality, health status and stress levels [19, 42–46].

#### Immunological variables

Based on previous studies [47] the following variables were used to assess humoral and cellular immune responses at different experimental stages; (1) heterophil/lymphocyte (H/L) ratio [14,48,49] was used as a cellular representative, as well as a haematological indicator of chronic stress. Elevated H/L ratios have previously been associated with high stress levels [49,50]; (2) the lymphoproliferative response to phytohemagglutinin-p (PHA-P) [14,51,52] was also used as a cellular representative and as an indicator of the pro-inflammatory potential of each bird; and (3) the primary antibody response against sheep red blood cells (SRBC) [14,53,54] was used as a humoral indicator of acquired immunity. The procedure and time schedule for each immunological test is summarised in Table 2. Analyses of the physiological measures were carried out blind to the treatment.

**Table 2:** Immune variables measured and time schedule for each test performed. T1, T2, T3 and T4 are times 1–4 respectively; CSTR/NSTR are the Cold Stress and No Stress treatments; ISCP refers the Intermittent Stressful Challenges Protocol.

Variable measured	Procedure	Sampling Time
**Measure of heterophil/ lymphocyte (H/L) ratio**	The left wing brachial vein of each bird was punctured to obtain one blood drop for smears. Leukocyte counts were obtained by analysing the blood smears stained with May Grünwald Giemsa using an optic microscope. The proportion of each leucocyte type was calculated over counts of 100 white cells per smear. Afterwards, the H/L ratio was calculated for each bird and sampling day.	1^st^ smear collection: Day 3 (T1), one day post exposure to CS/NS treatment.
		2^nd^ smear collection: Day 18 (T2), before exposure to ISCP.
		3^rd^ smear collection: Day 28 (T3), post ISCP exposure.
		4^th^ smear collection: Day 36 (T4) one week after ICSP exposure.
**Lymphoproliferative response to phytohemagglutinin-p (PHA-P) injection**	Each bird was injected with 0.05 ml of a PHA-P solution in phosphate saline buffer (1mg/ml) in the wing web (intradermal injection). The injection site was marked with a non-toxic permanent marker. Wing web thickness was measured just before and 24h after the PHA-P injection. The dermal swelling response was reported as the percentage increase in wing web thickness at the injection site. Measurements were recorded using a digital calliper (Biltema, Sweden) to the nearest 0.01 mm.	1^st^ Injection: On Day 7, five days post exposure to the CSTR/NSTR treatment. Inflammation was determined 24 h later.
		2^nd^ Injection: On Day 28, after birds were exposed to the ISCP. Inflammation was determined 24 h later.
**Primary antibody response against sheep red blood cells (SRBC)**	Birds were intraperitoneally injected with 0.05 ml of a 10% solution of (SRBC) to induce a humoral immune response. One week after the SRBC administration, 0.5 ml of blood from each bird was collected from the brachial vein opposite to the wing used in the PHA-P test. Syringes were filled with two drops of E.D.T.A K3 to avoid coagulation. Blood samples were kept in Eppendorf tubes placed on ice till centrifuged. Samples were centrifuged at 2500 rpm/15 min and the serum obtained was distributed in Eppendorf tubes and kept at -20°C until analysis. The antibody response was assessed with a microagglutination assay [53–56]. Antibody titters were reported as the Log_2_ of the highest dilution yielding significant agglutination.	Injection: On Day 28, after birds were exposed to the ISCP.
		*Due to a possible contamin-ation of the injected blood, a total of 24 chicks either died or were euthanized. Data were corrected to the actual group size to account for this.

#### Behaviour and use of space in home pens

Direct behavioural observations according to a standardised ethogram (Table 3, adapted from [57–58]) were conducted two times per week between 9:00–15:00h. The exception to this was week four, when the ISCP was applied, when observations were performed only once. A scan sampling approach was used in which the observer noted the number of birds performing each behaviour at the time of the scan [58–59]. The general activities included; resting, standing, walking, preening, eating, foraging and drinking.

**Table 3:** Ethogram used for the data collection.

Behaviour	Description
Eating	Pecking and/or eating at feeder.
Drinking	Pecking and/or drinking at drinker.
Foraging	Pecking and scratching in litter.
Resting	Lying down inactive or standing with closed eyes or head tucked under wing.
Standing	Standing inactive with open eyes (includes both alert and non-alert states).
Walking	Bird takes ≥2 steps forward.
Preening	Bird arranges or oils her feathers with her beak.

**Fig 3:**
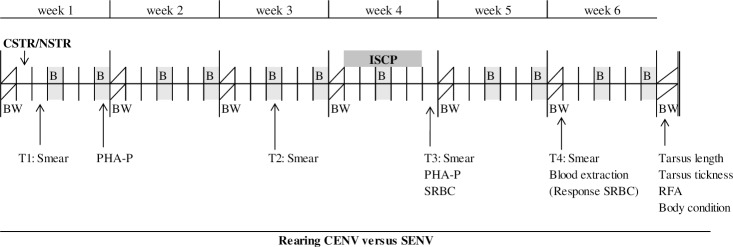
Chronogram of the study. Body weight (BW) was measured the first day of the study in order to balance groups accordingly and every week afterwards; birds were either assigned to a Complex Environment (CENV) or to a Simple Environment (SENV) as their rearing system; on day two of age, half of the birds from each environment were submitted to either a Cold Stress (CSTR) or a No Stress (NSTR) treatment; behavioural observations (B) were performed two times per week except for week four when the Intermittent Stressful Challenges Protocol (ISCP) was applied. The time of the measure of each immunological parameter (smears for accounting the heterophil/lymphocyte ratio (H/L ratio), Phytohemagglutinin-P injection (PHA-P) and Sheep Red Blood Cells injection (SRBC)) are indicated by arrows in the chronogram; at the end of the study we also collected tarsus weight, tarsus thickness and calculated body condition and Relative Fluctuating Asymmetry (RFA).

For birds in the CENV, we also noted their locations (on a perch, in the hide area, on the hide area platform, on a wooden block or on the floor) at the time of the scan. When a bird was observed perching, the perch level was also registered. A total of 20 scans/day were conducted across the eight experimental pens, divided into two morning and two afternoon blocks. Each block therefore consisted of five scans and these were performed in a random order across the eight pens. Since the pens looked different, the person doing the observations could not be blind to the environmental treatment. For a better overview, a detailed chronogram of all the treatments and tests performed during the study is shown in Fig 3.

### Statistical analyses

The GLIMMIX procedure in SAS V.9.3 (SAS Inst. Inc., Cary, NC) was used for the analysis of data in this experiment. Different distributions were used in the different analyses depending on the most appropriate fit for the data as detailed below. A compound symmetry matrix was used for those analyses with repeated measures over time. When necessary a Konward-Roger adjustment for the degrees of freedom was used [60]. The details of the specific analyses are given below.

#### Effect of cold stress/no stress treatment on internal body temperature

Firstly, we explored whether the CSTR/NSTR treatment affected the internal body temperature of the birds during the six hours of exposure. CSTR/NSTR treatment, time of exposure (0, 2, 4, 6 hours) and their interaction were included in the model as fixed effects. Time of exposure was included as a repeated measure and bird identity nested to pen as the subject. Data fitted into a gamma distribution. To validate that this was an acute stressor, the internal body temperature of the cold stressed chicks should be significantly lower than that of the non-stressed chicks and the absolute environmental temperature used should be at least that previously shown to be associated with cold stress effects on behaviour [61].

#### Effect of intermittent stressful challenges protocol on feeding behaviour

To investigate the effect of at least one of the stimuli on the birds, the effect of the repeated exposure to one hour of heavy metal music reproduced at 90dB on the number of birds feeding was explored. In this case CSTR or NSTR treatment, CENV or SENV treatment, day and time of exposure (30 and 15 min before music reproduction (no music), 0, 15, 30, 45, 60 min (during music reproduction) and after music was switched off at 75 and 90 min (no music)), were included in the model as fixed effects. Day was introduced as random and pen as subject. Data fitted a normal distribution after square root transformation.

#### Morphological and immunological indicators

For those variables that were taken only once during the experiment (tarsus length, tarsus thickness, body condition, RFA and antibody response to SRBC injection) we used a model that included CSTR/NSTR treatment, CENV/SENV treatment and their interaction as fixed effects. Pen was included as a random effect and bird identity nested to pen as the subject. For those variables which were taken repeatedly during the experiment (body weight, swelling response after PHA-P injection, H/L ratio) the model included CSTR/NSTR treatment, CENV/SENV treatment, age and their interactions as fixed effects. Age was introduced as repeated measure and bird id nested to pen as the subject. Variables fitting a normal distribution were (1) raw data from the tarsus thickness, (2) square root transformed data from the RFA (tarsus length and thickness) and the swelling response after PHA-P injection; (3) log2 transformed data from the antibody response to the injection of SRBC. Body weight, body condition and tarsus length fitted a gamma distribution and H/L ratio fitted a lognormal distribution.

#### Behavioural indicators

Regarding behavioural activity, the mean frequency of pullets performing each of the mutually exclusive behaviours was averaged first by observation round (mean of five scans/pen), and then by day and week. Data were corrected for the actual group size per pen at the time of the scan. Eating, drinking, foraging, preening, resting, standing and walking frequency fitted a normal distribution. CSTR/NSTR treatment, CENV/SENV treatment, week of age and their interactions were included in the model as fixed effects. Week of age was included as a repeated measure and pen as the subject.

#### Use of space

For the CENV treatment the mean frequency of birds on the floor or using the structures provided (perches, hide area, platform over the hide area or the wooden blocks) was calculated per scan and data were corrected for group size. The mean frequency of birds using each structure by observation round (mean of five scans/pen) day and week was calculated. Data for the use of the hide area and the floor followed a normal distribution, while data from the use of the perches, wooden blocks and the platform on the hide area were square root transformed. CSTR/NSTR, week of age and their interactions were included in the models as fixed effects. Week of age was introduced as a repeated measure and pen as the subject. Additionally, potential effects of CSTR/NSTR and week of age on the use of the different levels of the perches (1, 2, 3, 4) were explored. In this case we calculated the mean frequency of birds using each perch level per observation round (mean of five scans/pen corrected by group size) day and week. In this analysis we included CSTR/NSTR, week of age and perch level as fixed factors, as well as, CSTR/NSTR by perch level and week of age by perch level interactions. Week of age was introduced as a repeated measure effect and pen as a subject. Data were fitted to a normal distribution after square root transformation.

## Results

### Effect of acute cold stress on internal body temperature

**Fig 4:**
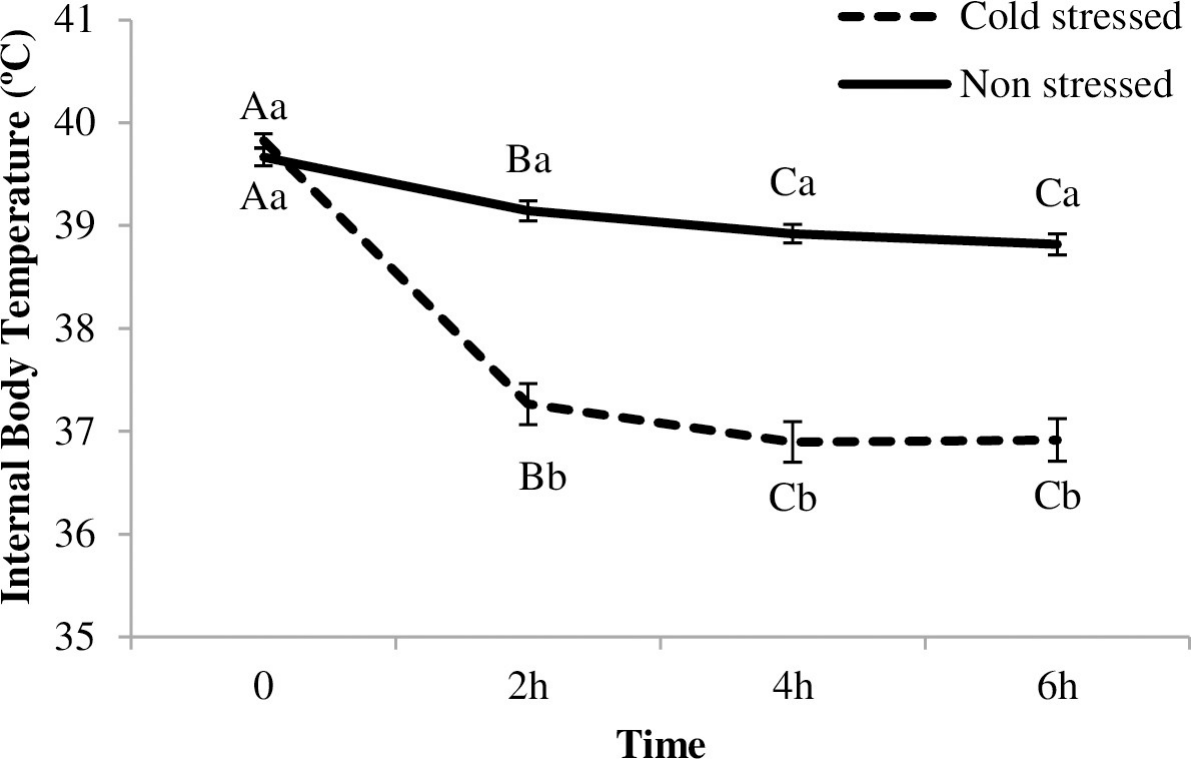
Internal body temperature for Cold Stress (CSTR) No Stress chicks (NSTR). Mean (± SE) internal body temperature for acute CSTR and NSTR chicks; temperature was registered in °C every 2h, at 0 (beginning of the cold stress), 2h, 4h, and 6h; ^(A-C)^ letters indicate differences over time within the CSTR or NSTR treatment; ^(a-b)^ letters indicate differences between CSTR and NSTR birds at the same time point.

There was a significant effect of the CSTR/NSTR treatment, time and their interaction (CSTR/NSTR treatment x time) on chicks’ body temperature (F_1,102_ = 63.3, p<0.0001; F_3,304_ = 263.26, p<0.0001; F_3,304_ = 94.64, p<0.0001 respectively). Body temperature decreased in both stressed and non-stressed birds. However, as shown in Fig 4, body temperature in CSTR birds decreased significantly more than in NSTR birds (average decrease of 0.8°C for NSTR vs. 2.9°C for CSTR).

### Effect of the intermittent stressful challenges protocol on feeding patterns

**Fig 5:**
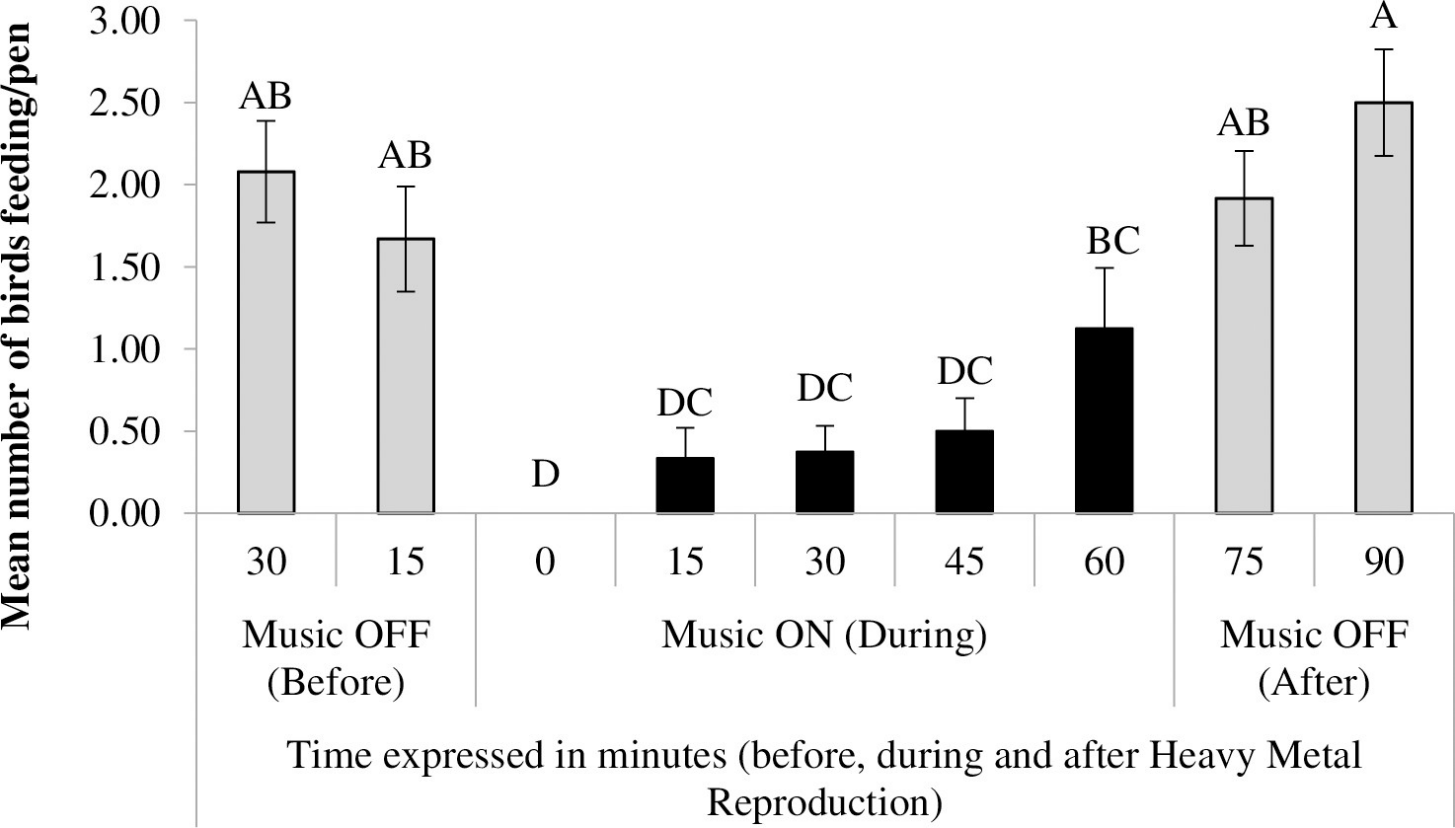
Number of animals feeding during application of the intermittent stressful challenges protocol (ISCP). Mean (± SE) number of animals feeding before (30, 15 min), during (0, 15, 45 and 60 min) and after (75 and 90 min) exposure to heavy metal music reproduced at 90Db; ^(A-D)^ letters indicate differences across time.

There was an effect of time (F_8,56_ = 17.16, p<0.0001) on feeding activity during the one hour exposure to heavy metal music in the ISCP. Fig 5 illustrates the differences across time on feeding activity. Birds drastically reduced their feeding activity when the music started. Feeding activity increased in the final 15 minutes of music exposure and returned to pre music levels after the music was switched off. No differences across days, CENV/SENV or CSTR/NSTR treatments were found (p>0.05).

### Body growth and other morphological indicators

**Fig 6:**
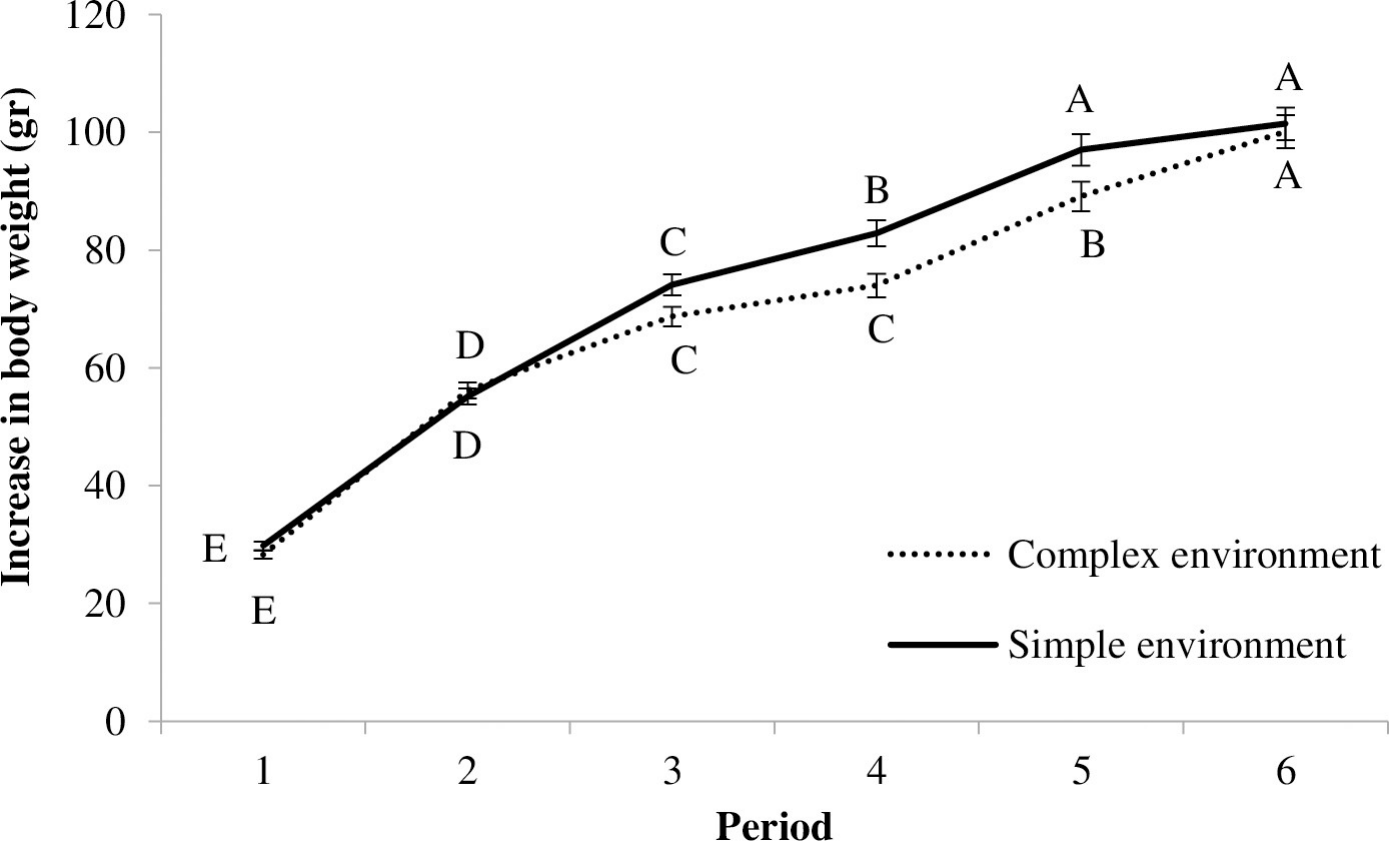
Increase in body weight estimated between two consecutive weeks. ^(A-E)^ letters indicate significant differences across time within each CENV or SENV treatment (p<0.05); there were no significant differences between CENV/SENV treatments at the same age period.

An effect of CENV/SENV treatment, age and their interaction was found when analysing body growth between consecutive weeks (F_1,92.64_ = 6.43, p = 0.01; F_4,410.2_ = 805.54, p<0.0001; F_5,410.2_ = 2.30, p = 0.04 respectively). Chicks reared in a CENV showed a slightly slower growth curve compared to those from SENV for age periods 3, 4 and 5 (Fig 6). However, birds from a CENV seem to compensate by growing faster and reached similar levels to SENV birds by the end of the study. No effects of the CSTR/NSTR treatment were detected for this variable (p>0.05).

At the age of six weeks, pullets reared in the CENV had a shorter tarsus and a lower body condition than those reared in the SENV (tarsus length; CENV: 59.01±0.42, SENV: 60.30±0.41, F_1,68_ = 5.08 p = 0.03; body condition (body weight/tarsus length); CENV: 7.64±0.07, SENV:7.87±0.07, F_1,68_ = 4.49, p = 0.04). No differences in tarsus thickness (F_1,68_ = 0.06, p = 0.81) or in RFA for tarsus thickness (F_1,69_ = 0.34, p = 0.56) were detected according to the complexity of the rearing environment. However, there was a tendency for a smaller RFA for tarsus length in the simple environment (CENV: 0.02±0.01, SENV: 0.01±0.009, F_1,68_ = 3.02, p = 0.09). No effect of CSTR/NSTR treatment or the CSTR/NSTR by CENV/SENV interaction were found for any morphological parameter (p>0.1).

### Immunological indicators

#### Heterophil/lymphocyte ratio

**Fig 7:**
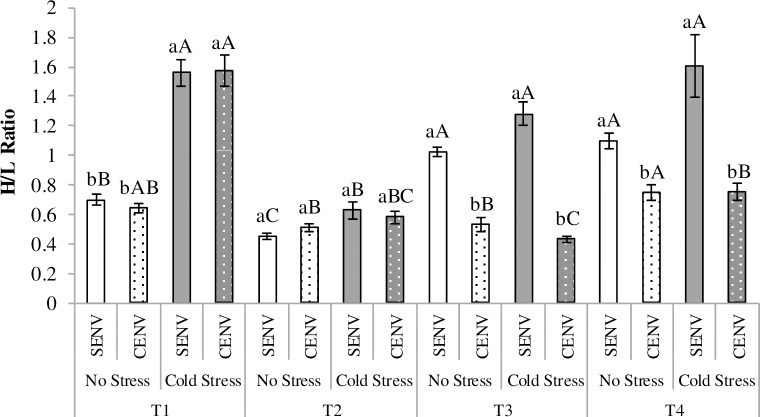
Heterophil/lymphocyte ratio evolution according to the different treatments. Mean (± se) Heterophil/lymphocyte (H/L) ratios in Cold Stress (CSTR) and No Stress (NSTR) birds kept in the Simple Environment (SENV) and a Complex Environment (CENV); NSTR treatment is represented by white bars, while CSTR by grey bars; SENV is represented by bars with a solid colour and CENV by bars with dots; ^(a-b)^ letters indicate differences between treatments at each sampling day and ^(A-C)^ letters indicate differences between the four different sample points; (T1) 2 days after hatching (directly after the exposure to the CSTR/NSTR treatment); (T2) 18 days after hatching; (T3) 28 days after hatching (just after the 5-days of exposure to the ISCP); (T4) 36 days after hatching (one week after the exposure to the ISCP).

The results of the H/L ratio are reported in Table 4 and reveal a significant effect of the three way interaction CSTR/NSTR by CENV/SENV and age (Fig 7). In this case, CSTR birds showed a higher H/L ratio than NSTR at day three of age (T1), directly after exposure to CSTR/NSTR treatment, suggesting high stress [49–50]. By T2, the H/L ratio in CSTR birds had decreased to levels similar to that of the NSTR birds. No effect of the rearing environment was observed in either of these first two sampling points.

**Table 4:** Results from the statistical analyses regarding heterophil/lymphocyte (H/L) ratio and the swelling response after the phytohemagglutinin-P (PHA-P) injection, expressed as percentage of inflammation.

	H/L Ratio			Percentage of Inflammation		
Effects	F-value	DF	p-value	F-value	DF	p-value
CSTR/NSTR	78.87	1,96.15	<0.0001	20.53	1, 73.32	<0.0001
CENV/SENV	115.11	1,96.15	<0.0001	15.32	1, 73.32	0.0002
Age	93.39	1,253.8	<0.0001	249.22	1, 80.55	<0.0001
CSTR/NSTR x CENV/SENV	6.67	1,96.15	0.01	39.95	1, 73.32	<0.0001
CSTR/NSTR x Age	34.24	1,253.8	<0.0001	12.25	1, 80.55	0.0008
CENV/SENV x Age	47.08	1,253.8	<0.0001	0.11	1, 80.55	0.74
CSTR/NSTR x CENV/SENV x Age	2.59	1,253.8	0.05	9.70	1, 80.55	0.003

Interestingly at T3, directly after exposure to the ISCP, birds kept in the SENV treatment had a significantly raised H/L ratio irrespective of whether they had been submitted to the CSTR or to the NSTR treatments early in life (Fig 7). This increase persisted and at T4 birds from the SENV had higher H/L ratios compared to birds in the CENV.

#### Lymphoproliferation to PHA-P injection and antibody response to SRBC

**Fig 8:**
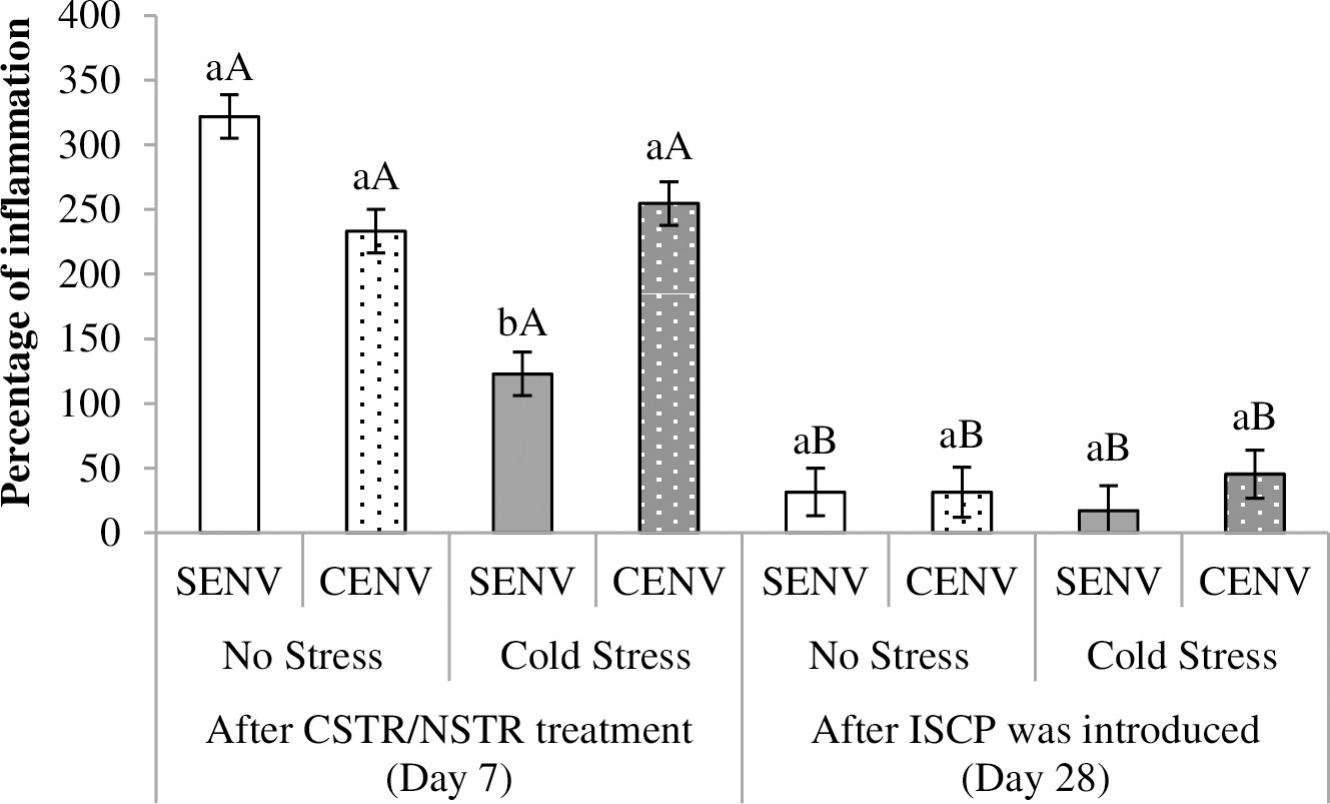
Mean (± SE) percentage of inflammation measured after PHA-P injection. ^**(**a-b)^ letters indicate differences between different treatments at each injection time i.e. Day 7 of age (five days after Cold Stress (CSTR)/No Stress (NSTR) treatment) and Day 28 of age (after the ISCP was applied); ^(A-B)^ letters indicate differences across time within the same treatment; NSTR treatment is represented by white bars; CSTR by grey bars; SENV is represented by bars with a solid colour; CENV by bars with dots.

Results from the swelling response after PHA-P injection are reported in Table 4. Similar to the results for the H/L ratio, the results revealed a significant effect of the triple interaction CSTR/NSTR by CENV/SENV and age (Fig 8). Birds that were CSTR and reared in a SENV showed the lowest swelling response after the first PHA-P injection, some days after the exposure to the CSTR/NSTR treatment and immediately after the ISCP was applied. All groups were found to have significantly diminished inflammatory response after being submitted to the ISCP.

An effect of CSTR/NSTR treatment was found when analysing the antibody response to SRBC (F_1,57_ = 5.03, p = 0.03). CSTR birds had a significantly lower production of antibodies than NSTR birds (CSTR: 2.40±0.32, NSTR: 3.38±0.32). No effect of CENV/SENV or of the interaction was found (p>0.05).

### Behaviour in the home pen

**Fig 9:**
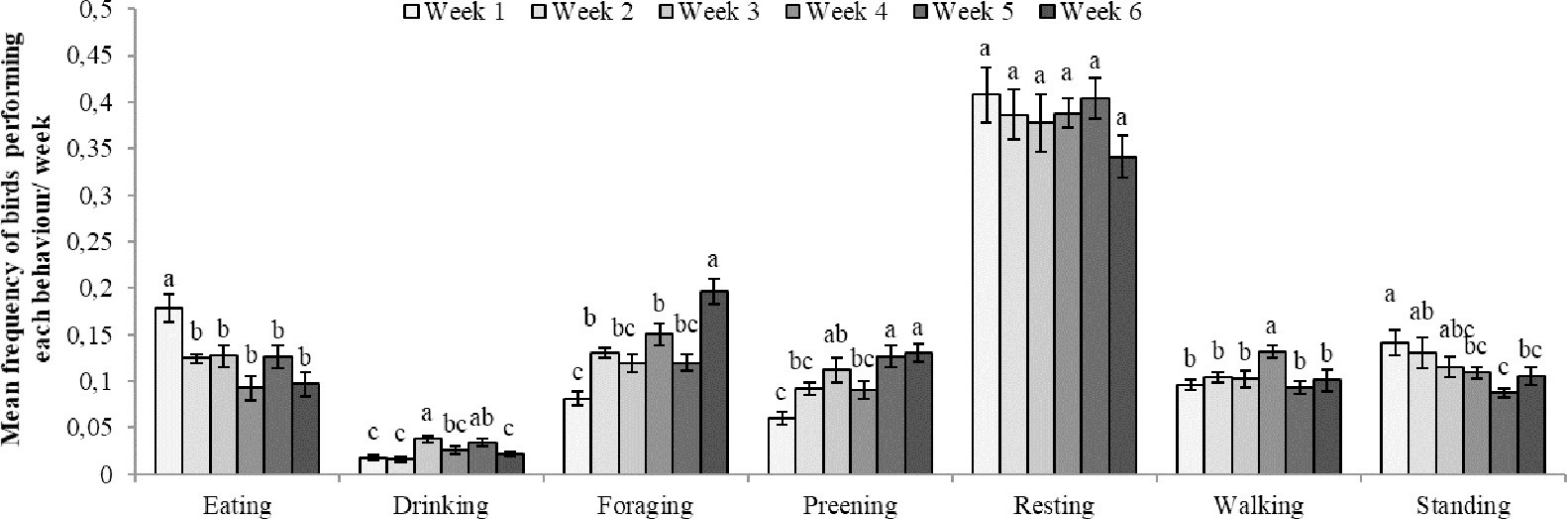
Mean (± SE) frequency of birds performing each behaviour by week. ^(a-c)^ letters indicate significant differences across weeks for eating, drinking, foraging, preening, resting, walking and standing.

Chicks in the CENV rested more, as well as they stood and walked less than chicks in the SENV (resting: F_1,5_ = 17.67, p = 0.008, CENV: 0.43±0.01 SENV:0.34±0.01; standing: F_1,5_ = 8.12, p = 0.04, CENV: 0.1±0.005, SENV:0.13±0.007; walking: F_1,5_ = 19.62, p = 0.007, CENV: 0.09±0.004, SENV: 0.12±0.004). No effect of CENV/SENV was detected for any of the other behaviours (preening, eating, foraging and drinking, p>0.05 in all cases). No effect of the CSTR/NSTR treatment was observed for any of the behaviours studied. An effect of week of age was found for eating (F_5,35_ = 6.70, p = 0.0002), foraging (F_5,35_ = 13.73, p<0.0001), preening (F_5,35_ = 11.60, p<0.0001), standing (F_5,35_ = 6.87, p = 0.0001), walking (F_5,35_ = 6.00, p = 0.0004) and drinking (F_5,35_ = 6.30, p<0.0003), (Fig 9). No significant differences on resting were observed across time (p>0.05).

### Use of space

**Fig 10:**
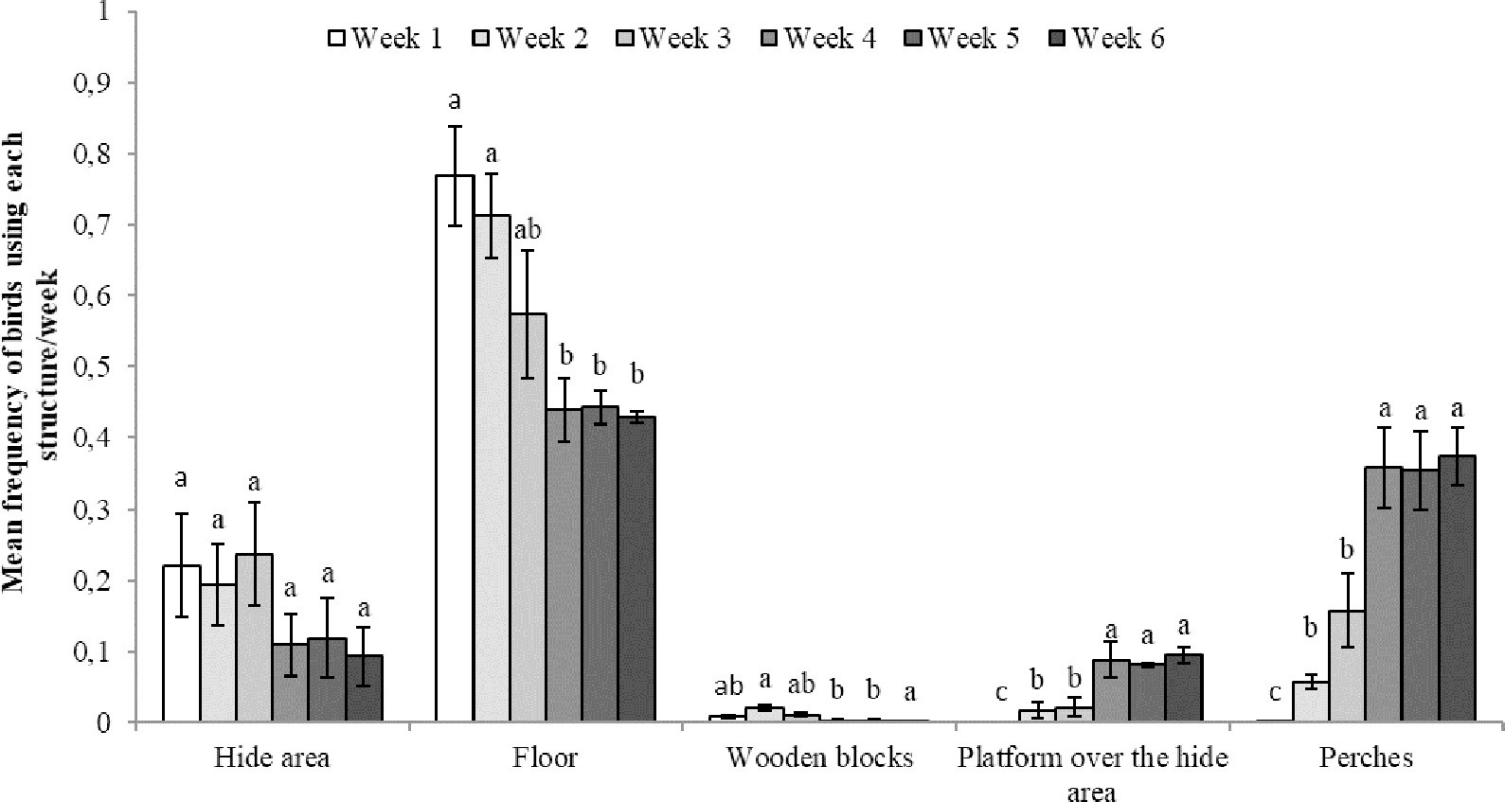
Use of different areas in the complex environment treatment. Mean (+ SE) frequency of birds using the different areas of the pen by week in the CENV treatment; ^(a-d)^ letters indicate differences in the use of the structures (P<0.05) across weeks.

**Fig 11:**
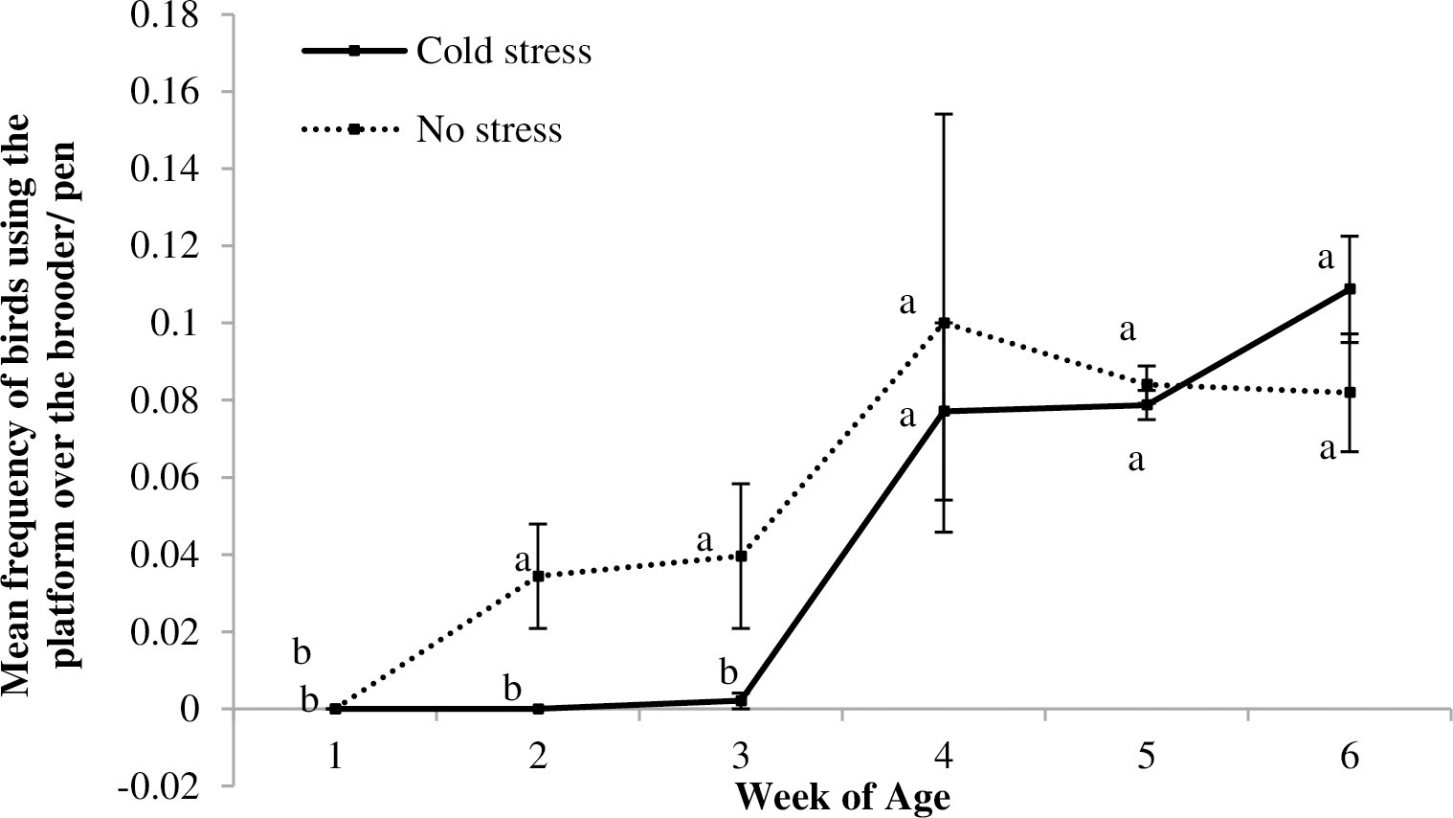
Use of the platform over the hide area for CSTR and NSTR birds. Mean (+ SE) frequency of birds using the platform over the hide area for the Cold Stress (CSTR) and No Stress (NSTR) treatments across different weeks; ^(a-b)^ letters indicate differences within each treatment across weeks; no differences among treatments were observed in post hoc comparisons regarding each observed week.

**Fig 12:**
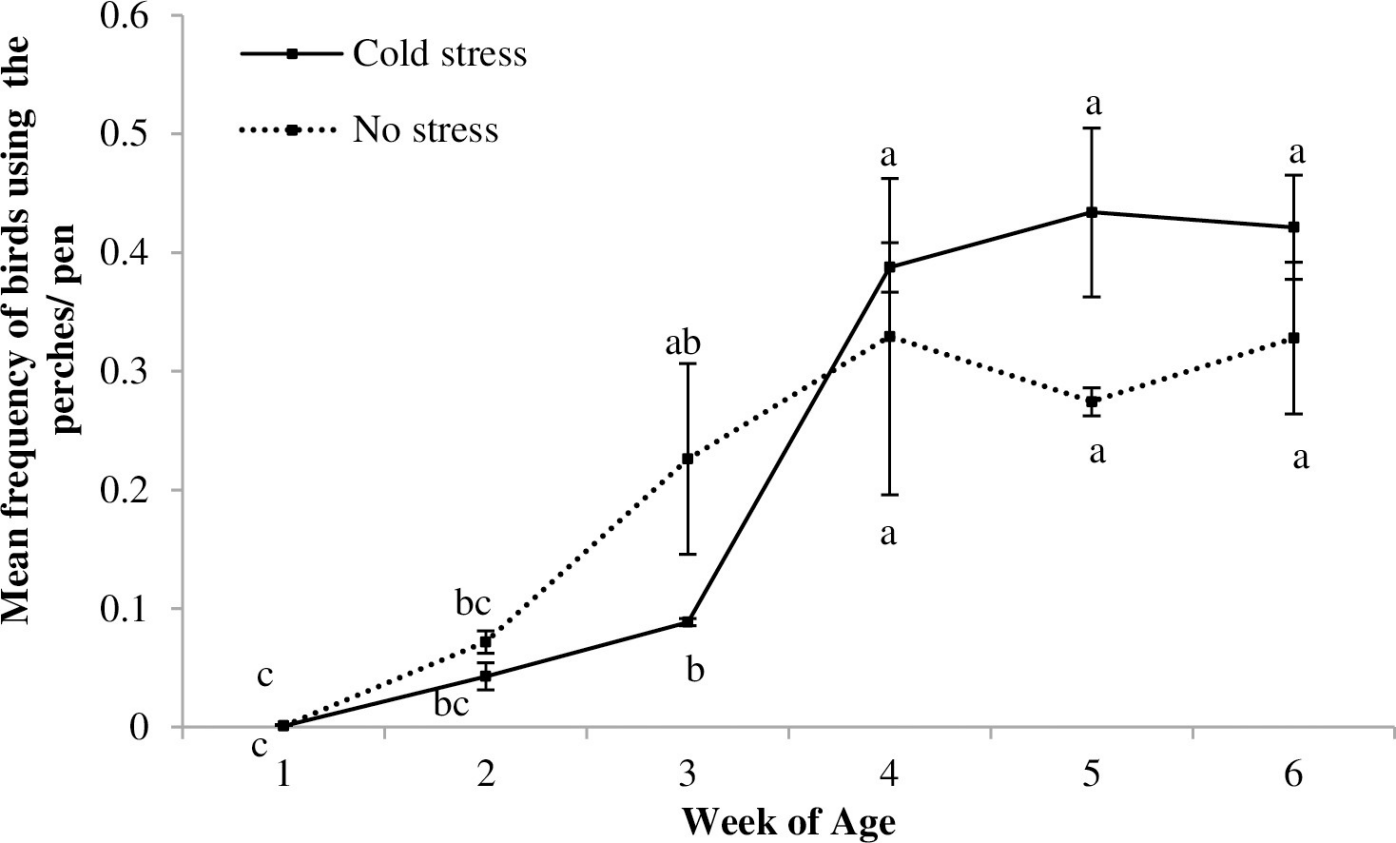
Use of perches by CSTR and NSTR birds. Mean (+ SE) frequency of birds perching for the Cold Stress (CSTR) and No Stress (NSTR) treatments across different weeks; ^(a-b)^ letters indicate differences within each treatment across weeks; no differences among treatments were observed in post hoc comparisons regarding each observed week.

An effect of week of age was detected in the CENV pens, with birds using the floor less and the raised areas more with increasing age (Floor: F_5,10_ = 9.91, p = 0.001; wooden blocks: F_5,10_ = 6.22, p = 0.007; hide area: F_5,10_ = 3.36, p = 0.05; platform over hide area: F_5,10_ = 50.26, p<0.007; and perches: F_5,10_ = 59.99, p<0.0001; Fig 10). No effect of CSTR/NSTR treatment was observed, but there was an interaction between CSTR/NSTR and week of age for the use of the platform over the hide area (F_1,5_ = 6.41, p = 0.006; Fig 11) and a tendency for the same interaction in the use of perches (F_5,10_ = 3.15, p = 0.06, Fig 12).

We also found an effect of week of age (F_5,15_ = 18.56, p<0.001), perch level (F_3,6_ = 21.80, p = 0.0013) their interaction (week of age by perch level) on the mean frequency of use of perches (F_15,45_ = 5.41, p<0.0001). Birds started by using the lower perches, but they used the higher perches more often as they got older. No effect of CSTR/NSTR or the interaction CSTR/NSTR by week of age was observed.

## Discussion

Our main results support the hypothesis that providing chicks with a more complex environment can help them improve some immunological and behavioural responses related to early stressors, as well as preparing them for future challenges. More precisely, we found that a CENV can ameliorate the effects of CSTR, allowing birds to express a higher pro-inflammatory response against PHA-P than their conspecific reared in a SENV (the first time PHA-P was administrated). Although there were no beneficial effects of the CENV initially, when analysing the H/L ratio during the three weeks directly after the CSTR there was a later buffering effect. Birds in the CENV had a lower H/L ratio following exposure to the ISCP compared to those reared in the SENV. No stress alleviating effects of the CENV environment after the ISCP were seen on birds’ pro-inflammatory response to PHA-P or in their antibody response against SRBC. Additionally, but no less important, we found that the early CSTR experience, rather than improving chicks’ capacity to cope with future challenges, actually seemed to make them more sensitive or vulnerable to them. This is based on their lower humoral response (antibody production against SRBC) and their slower start to use the elevated structures in the pen, such as the platform over the hide area and the perches. Although not all the consequences associated with the CENV are necessarily related to an immunological, behavioural and morphological improvement, the combined results from our investigation suggest that provision of a CENV early in life may contribute to improved welfare for poultry along different stages of their ontogeny.

### Effect of CSTR/NSTR and CENV/SENV on physiological, morphological and immunological indicators

Considering that exposure to low temperatures is associated with increased energy needs for warmth, a reduction of the abdominal fat content was expected in pullets experiencing cold stress [62–64]. Consequently, CSTR birds might have been expected to have lower body weights, slower growth rates or poorer body condition than NSTR birds. However, we did not detect any difference between birds for any of these variables. The lack of differences could be due to the relatively short period of time (6h) during which the stressor was applied, or because birds may have recouped any loss of fat since food was provided *ad-libitum* once they returned to the pens. Additionally, we did not detect differences in the RFA of tarsus length and tarsus thickness. This would indicate that CSTR/NSTR birds probably experienced similar levels of stress, which is in accordance with the findings of Campo et al. [65].

We observed that CENV birds showed a slightly slower growth curve in weeks four and five than birds reared in a SENV (Fig 6), but these differences disappeared by week six resulting in a similar weight for birds in both treatments. Birds reared in a CENV also had a slightly shorter tarsus length and lower body condition. These results might be a consequence of a higher level of exercise performed by birds in the CENV where there were perches and a platform. Increased exercise may be consistent with the fact that birds in the CENV showed more resting behaviour, although the furnishing in the CENV may also have made it easier for birds to rest undisturbed for longer.

Different scenarios were found for the different immunological indicators, the different stress sources and the time elapsed between stressors. The H/L ratios measured the day after birds were exposed to the acute cold stress were affected by the CSTR, as values were higher in CSTR birds than in their NSTR counterparts (Fig 7, T1). Previous research by Shinder et al. [66] indicated that exposing birds to cold stress (15°C during 3h) for the first time at three and four days of age resulted in a significant increase in plasma corticosterone concentration. The maintenance of an elevated plasma glucocorticoid concentration, induced by repeated exposure to stressful conditions, is known to reduce lymphocyte numbers and increase the number of neutrophils/heterophils [67–68]. Our results indicate that acute cold stress during 6 h on only one day was sufficient to induce a physiological alteration affecting leukocyte distribution, similar to that observed with longer chronic stress exposures. In practice, such a stressor can occur when young chicks are transported from the hatchery to the rearing house.

No effect of housing environment (CENV/SENV) on H/L ratio was detected at T1. However at that time, birds had been exposed to the environment for less than 24h. By the second time this variable was evaluated, birds were already 18 days of age (T2). We found that CSTR birds had significantly decreased their H/L ratio, reaching the values of their NSTR companions, which may indicate a time-dependent recovery phenomenon in this variable. Opposite to what we expected, no effect of the CENV was observed in T2, which may also indicate that recovery after CSTR was not influenced by the CENV. Directly after exposure to the ISCP (28 day old chicks-T3) we found a significant increase in H/L ratio, but only for birds reared in a SENV and independently of whether they had been exposed to CSTR early in life (Fig 7). It could be hypothesised that the CENV might have prepared the birds to cope with new challenges (such as those used in the ISCP) and this is why birds reared in the CENV showed a reduced H/L ratio, indicative of lower levels of circulating stress response mediators. The acute CSTR applied in the first days of life seemed not to have had long lasting effects according to the results of the third sampling for this particular variable. Interestingly, the week after exposure to the ISCP (36 days old-T4) birds from all treatments had significantly increased their H/L ratio compared to the values observed before the ISCP (T2), but birds from the CENV treatment still showed significantly lower H/L ratios than those reared in the SENV. Increased environmental complexity, therefore, does have a positive effect on this immunological parameter.

Similar to our findings, increased H/L ratios have been observed in birds submitted to sources of stress such as fasting and frustration of feeding [69], continuous lighting [65] or 1h of noise reproduced at 90dB [70], which confirms that these types of environmental stressors have negative effects on the immune system of domesticated avian species. Birds reared in the SENV had an immediate physiological stress response to the ISCP, inducing an elevation of the H/L ratio in T3, and they maintained it in T4. Given time, birds reared in the CENV seemed to cope with the ISCP since one week later they had only a small increase in the H/L ratio (lower than the increase observed in SENV birds).

An ameliorating effect of CENV was also detected in the response to the PHA-P injection at seven days of age (five days after the cold stress treatment). By this time CSTR birds reared in a CENV showed a swelling response similar to that show by NSTR birds reared in either a SENV or in a CENV. In addition, this response was higher than that observed for their CSTR counterparts reared in a SENV (Fig 8). These results give additional support to our hypothesis that increasing environmental complexity is beneficial for the immune responses of stressed animals. Furthermore, it may provide information regarding how strong or long lasting the influence of CSTR may be. Based on the conditions used in this study, our results indicate that even five days after exposure to an acute cold stressor, the birds in the SENV were still affected by the consequences, displaying a diminished global pro-inflammatory potential.

However, by the second time PHA-P was measured (28 days old chicks, just after the ISCP) no ameliorative effect of CENV was observed. Even though by this time birds from the CSTR treatment and reared in a SENV were the ones showing the lower PHA-P response, all groups had a similar reduction in their inflammatory response. This could be explained in two different and not necessarily mutually exclusive ways. On one hand, it was the second time that birds were receiving the PHA-P injection, and second exposure to these types of antigens could induce lower responses. On the other hand, the effects of ISCP exposure on the birds’ immune system may have been so great that it resulted in birds decreasing their response to a minimum, regardless of any environmental enrichment effects.

This repeated challenge issue is not applicable for the SRBC antibody response, since it was only applied once during the development of this experiment, after exposure to the ISCP. We observed that CSTR birds had a significantly lower antibody response than NSTR birds, which suggests that by the end of the experiment those birds exposed to CSTR early in life had their humoral immune responses altered. Similarly, Cichon et al. [71] found that mice showed lower antibody production against SRBC after a chronic exposure to CSTR. Svensson et al. [72] has also demonstrated that cold temperatures suppress antibody production in blue tits (*Parus caeruleus*). No ameliorating effect of the CENV was observed in our study regarding this variable.

Considering the results of our study, it is particularly informative to focus attention on the time points when the immune-related variables were sampled simultaneously. These experimental points were T3 (H/L ratio, PHA-P response and induction of SRBC response) and T4 (H/L ratio and SRBC response quantification). At T3, which was after the ISCP, birds showed diminished and homogeneous pro-inflammatory potential, together with an elevated H/L ratio if they were reared in the SENV. This is a potentially dangerous scenario. These birds had diminished pro-inflammatory potential and are also showing haematological indicators of a chronic stress response. Exposure to pathogens at this particular time, or near to it, would have been energy demanding and involve non-solved complications for the animals. Which may, unfortunately, have been what happened in this study for some birds. With regard to T4, and important to take into account in order to understand the situation at this point in time, is that birds had already been induced to provide a humoral antibody-mediated response in T3 (just after the ISCP). The diminished antibody response observed in the birds previously exposed to acute CSTR could therefore be indicating a cumulative effect of stressors along their ontogeny, because the non-stressed animals show no reduced antibody titres. The low H/L ratio at this time point supports (as previously mentioned) a better stress coping by CENV reared birds. This last point does not invalidate the fact that humoral responses were globally affected in CSTR and ISCP exposed groups.

Nazar and Marin [14] reported that non-enriched/non-stressed quail had the lowest swelling response after PHA-P injection and the highest H/L ratio in comparison to the other treatments (enriched/stressed, non-enriched/stressed, non-enriched/non-stressed). Although, our results are quite similar regarding the pro-inflammatory response (PHA-P) we were not able to detect any ameliorative effect of CENV on the humoral component of the immune function (anti-inflammatory responses). One possible explanation for the differences across studies may be that in ours, half of the birds were submitted to two sources of stress (CSTR and ISCP), while in the study of Nazar and Marin [14] all birds were submitted to a single chronic stressor (15 min of daily restraint). An important question to address in future research, therefore, is whether the difference in the humoral response of CSTR birds was produced just after CSTR or, later on, after the additional exposure to the ISCP due to an accumulative effect of stressors.

### Effects of CSTR/NSTR and CENV/SENV on behaviour and use of space

No effects of CSTR were observed for the different behaviours analysed. This may reflect a lack of power in the study for those behaviours analysed at pen level. However, chicks reared in a CENV rested more and were observed to stand and walk less. Some differences in behavioural variables between the SENV and the CENV were expected due to the different nature of the resources provided. For example, previous research suggests that an increase in environmental complexity in the form of perches or cover decreases the number of disturbances from conspecifics and increases time spent resting [22–25]. Resting is important, especially for young animals [73], as it influences growth, energy conservation, tissue restoration and coping abilities [74,75]. If the provision of a CENV allows birds to rest more, it is probable that it also favours a better recovery after stressful events. This hypothesis is consistent with the part of our results regarding the ameliorative effect that the CENV had on some of the immunological variables, such as the first PHA-P injection and the H/L ratio after exposure to the ISCP. From a behavioural point of view, the provision of a CENV may have helped birds to cope better with new challenges also of a different nature. For example, provision of a CENV designed according to birds’ behavioural needs (e.g. favouring their escape responses) would provide chicks with a certain possibility to control their environment, which might be important to lower their stress response [6].

Interestingly, in a parallel study performed with the same chicks we found that birds from the CENV remained more optimistic after being submitted to the ISCP than those reared in a SENV [75]. This may indicate that birds from the CENV had a more positive affective state than those reared in a SENV, which could have helped counteract some of the stress-related deleterious effects. Previous studies in rats have already suggested that pessimism may make individuals more susceptible to stress [76] which seems consistent with the combined results found in our studies.

The Red Jungle fowl uses high places for rest and escape from ground predators and this preference for higher perches can still be observed in domestic lines [33,77–82]. Our results are in accordance with this. As the birds’ physical abilities and strength increased with age, they tended to spend more time on the higher perch and less time on the lower structures (Fig 10, Fig 11 and Fig 12). Resting as far as possible from the ground may give birds a greater sense of security [33,78,83] and may also have helped them recover after exposure to stressors.

An effect of the interaction between CSTR/NSTR treatment and week of age was found when analysing the use of the platform over the hide area and the use of perches. Our results indicate that CSTR birds had a slower development in their pattern of use of these elevated structures compared to NSTR birds (Figs 11 and 12). However, in the case of the use of perches, probably due to the low number of replicates, the post hoc results were inconclusive. According to the adaptive plasticity theory, the input individuals receive during their development may have long lasting effects on the modulation of their phenotypes [5]. Following this argument, we speculate that CSTR birds could have developed a phenotype that expresses more anxiety-like behaviour due to the input they received at an early age and that this led to them being more fearful in the exploration of their environment. Supporting this, and reported in the previously mentioned parallel study by Zidar et al. [75], CSTR birds were faster to first step in a detour social reinstatement test than NSTR birds after being exposed to the ISCP, which may be indicative of higher anxiety-like behaviour. It has also been shown that CSTR birds have longer tonic immobility duration, indicating higher fear levels [50]. At least in the conditions of this study, CSTR early in life seems to have resulted in a phenotype that was less well adapted to exploiting the potential of the CENV.

## Conclusions

This study demonstrates that environmental complexity during rearing may not only help to ameliorate the consequences of early stressful events, but may also help birds to cope better with future challenges. Even though not all the immunological or behavioural stress impaired responses were mitigated by the provision of a CENV, some of them were improved when compared with the responses observed in birds reared in a SENV. We could interpret our results in the context of the adaptive plasticity theory. The different inputs received by the birds in early life (cold stress or environmental complexity) would have differentially affected them, configuring potentially different phenotypes. Birds with a CENV as an input may have a better physiological response against stress than birds reared in the SENV. This would suggest that the wider possibilities to control their environment, and their physiological responses, for example by resting in a quiet area when needed or having higher activity possibilities, would mean that the stress affected them less or made them better able to develop stress coping strategies. Additionally, we determined that acute cold stress during early life may have short and long lasting effects depending on: i) the variable analysed, ii) whether there is a series of different inputs during ontogeny (e.g. an additional challenge such as an ISCP) and iii) the characteristics of the rearing environment. Given that even under good husbandry conditions, the rearing of laying hen pullets is never going to be stress-free, this research has contributed to increasing our understanding about why the provision of a complex environment is crucial for improving their welfare.
